# Study on Calcination Characteristics of Diaspore-Kaolin Bauxite Based on Machine Vision

**DOI:** 10.3390/molecules29163813

**Published:** 2024-08-11

**Authors:** Longjiang Li, Jun Liu

**Affiliations:** 1 College of Mining, Guizhou University, Guiyang 550025, China; liuj28050@sanygroup.com; 2 National & Local Joint Laboratory of Engineering for Effective Utilization of Regional Mineral Resources from Karst Areas, Guiyang 550025, China; 3 Guizhou Key Laboratory of Comprehensive Utilization of Non-Metallic Mineral Resources, Guiyang 550025, China

**Keywords:** bauxite, calcination characteristics, machine vision, color feature extraction

## Abstract

D-K-type bauxite from Guizhou can be used as an unburned ceramic, adsorbent, and geopolymer after low-temperature calcination. It aims to solve the problem where the color of the D–K-type bauxite changes after calcination at different temperatures. Digital image processing technology was used to extract the color characteristics of bauxite images after 10 min of calcination at various temperatures. Then, we analyzed changes in the chemical composition and micromorphology of bauxite before and after calcination and investigated the correlation between the color characteristics of images and composition changes after bauxite calcination. The test results indicated that after calcining bauxite at 500 °C to 1000 °C for 10 min, more obvious dehydration and decarburization reactions occurred. The main component gradually changed from diaspore to Al_2_O_3_, the chromaticity value of the image decreased from 0.0980 to 0.0515, the saturation value increased from 0.0161 to 0.2433, and the brightness value increased from 0.5890 to 0.7177. Studies have shown that changes in bauxite color characteristics are strongly correlated with changes in composition. This is important for directing bauxite calcination based on digital image processing from engineering viewpoints.

## 1. Introduction

Bauxite from Guizhou features rich resources, extensive reserves, and high grades [1]. At present, there are 13 mining areas of bauxite in Guizhou, and the deposit is mainly of sedimentary type, with a total reserve of ~326.94 million tons [2]. Generally, for the single ore, the Guizhou bauxite mining area mainly comprises the lower Cambrian and Carboniferous, Permian, and Trias periods. The bauxite layer in the upper or lower fracture of the main seam of the local area has small lenticular orebodies in earthy bauxite deposits, which are different types of dense bauxite ore [3]. There are two main processing methods for D-K bauxite raw materials: high temperature and low temperature. After high-temperature calcination, bauxite is mainly used to prepare refractory materials. In contrast, after low-temperature calcination, the calcined bauxite at low temperature is mainly used to produce adsorbents, unburned ceramics, geopolymers, etc. [4]. During the low-temperature calcination test of D-K bauxite from Xiuwen District, Guizhou Province, bauxite exhibited distinct color changes after calcination at various temperatures.

Due to variations in color characteristics, in this research, we applied machine vision and digital image processing technology to the calcination process of bauxite and replaced human eye observation with machine vision [5]. In recent years, color feature extraction has been widely used in mining and metallurgy based on machine vision technology [6,7]. Chen Qing [8] et al. directly estimated the concentration grade by extracting the color features of mineral flotation froth. Lv Shuo [9] et al. studied a new pellet grade evaluation system by extracting the ore phase characteristics of pellets. Yuan Hui [10] used the gray correlation method to extract the four image characteristic parameters of asphalt mixtures and mineral aggregates, providing a new evaluation index for asphalt mixtures and mineral aggregate gradation. Su Buxin [11] proposed a new method for the quantitative analysis of sinter mineral composition with higher recognition accuracy by extracting texture features from the microscopic images of the sinter and using knowledge base rules for classification. By combining fuzzy C-means clustering and the back-propagation neural network, Zhou Houwei [12] developed an online system that can automatically detect FeO content in the sinter in real-time.

Based on the above studies, we used detection methods such as X-ray diffraction (XRD) pattern analysis, X-ray fluorescence spectroscopy (XRF), scanning electron microscopy (SEM), and Fourier infrared spectroscopy (FT-IR) to analyze the microscopic evidence for compositional changes in bauxite under calcination at various temperatures. Then, by extracting the color characteristic parameters of bauxite images after calcination at various temperatures [13], the bauxite calcination effect can be quickly estimated to deal with an extensive waste of resources in the process of low-temperature calcination of bauxite due to over-burning and under-burning [14]. At present, the detection of the bauxite calcination effect mostly relies on manual judgments based on the experience of on-site technicians or is performed by using chemical methods and large-scale instruments. These methods have complicated, time-consuming, and costly operations. The new method of detecting bauxite calcination effects based on machine vision can reduce adverse effects such as chemical detection on bauxite calcination, lagging guidance at low temperatures; can reduce production costs; and can improve calcination efficiency. This is extremely important for improving the utilization rate of bauxite resources and building an energy-saving and environmentally friendly society.

## 2. Results and Discussion

### 2.1. Analysis of the Properties of Experimental Materials

**Figure 1:**
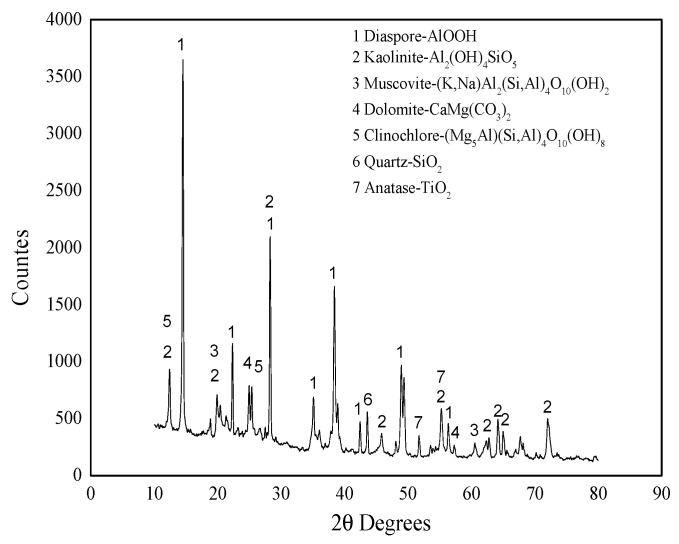
XRD pattern of bauxite raw material.

**Figure 2:**
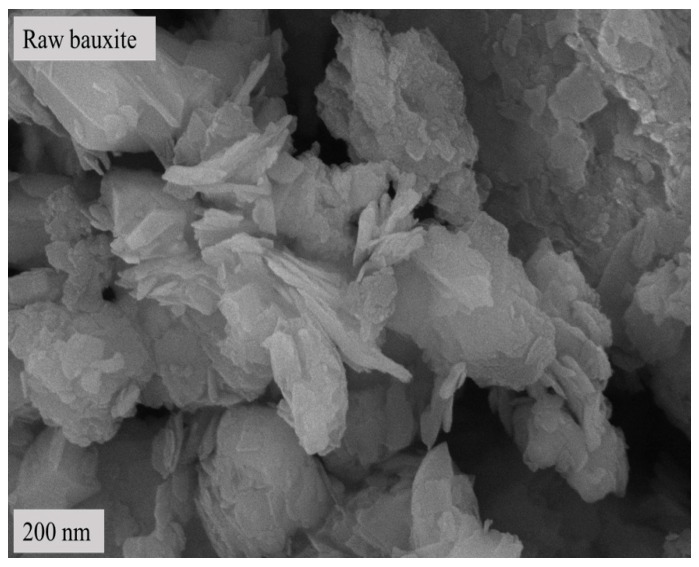
SEM image of bauxite raw material.

Table 1 shows the results of the X-ray fluorescence spectrum analysis of raw materials. The results of XRD pattern analysis are shown in Figure 1, and the scanning electron microscope image is shown in Figure 2.

Table 1 shows that the main component of the bauxite raw material is Al_2_O_3_, with a content of 60.39%, offering appropriate application potential. Figure 1 and Figure 2 indicate that the main component of the bauxite raw material is diaspore (AlO(OH)), and the bauxite raw material shows a well-defined layered structure according to SEM.

**Figure 3:**
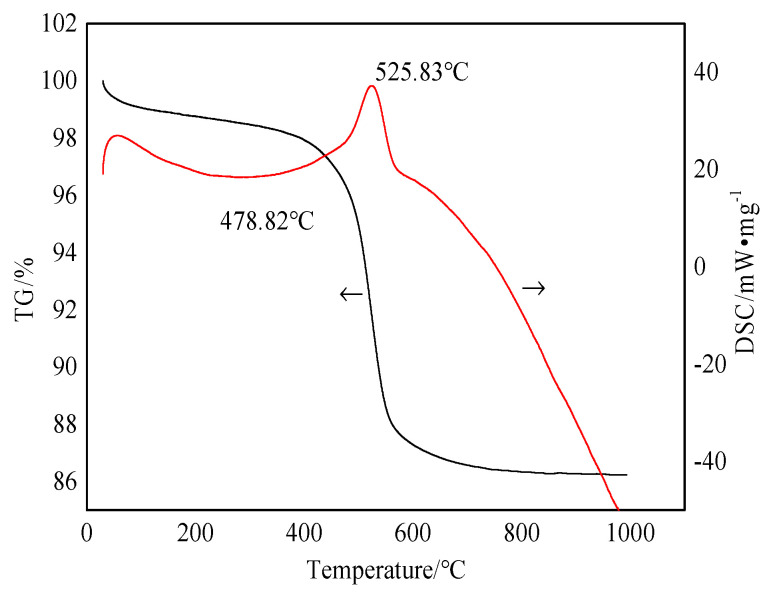
TG–DSC curve of bauxite raw material.

Figure 3 shows the differential scanning calorimetry and thermogravimetric analysis (TG–DSC) curves of the bauxite raw material.

Figure 3 shows that the bauxite is heated from room temperature to 1000 °C, with a total weight loss rate of about 13.768%. There is a small endothermic peak at the 0–100 °C section on the DSC curve. For this reason, the adsorbed water in the bauxite is evaporated before 100 °C. At 200–400 °C, the curve displays a series of tiny endothermic peaks caused by the interlayer water removal of layered silicate minerals (kaolinite, chlorite, and muscovite). When the calcination temperature rises to 400–600 °C, bauxite displays a noticeable weight loss in this temperature range. The DSC curve exhibits a strong endothermic peak. Combining these with the XRD analysis results shows that this is due to the removal of excess structural water from diaspore and kaolinite in the bauxite under this temperature range [15,16]. The bauxite slowly becomes weightless at 600–1000 °C, which is caused by crystal precipitation. According to the degree of difficulty, the degree of dehydration may be ranked as the dehydration of free water, weakly bound water on the surface, and chemically adsorbed bound water [17,18]. The chemical reactions are shown in Formulas (1)–(3):

(1) $$2AlOOH\to Al_{2}O_{3}+H_{2}O$$

(2) $$Al_{2}O_{3}\cdot H_{2}O\to Al_{2}O_{3}+H_{2}O$$

(3) $$AlSi_{2}O_{5}{\left(OH\right)}_{4}\to Al_{2}O_{3}\cdot 3SiO_{2}+2H_{2}O$$

### 2.2. XRD Pattern Analysis

**Figure 4:**
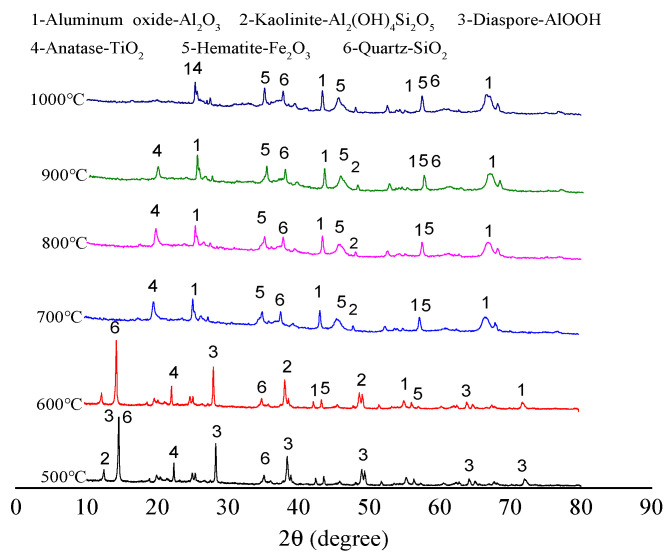
XRD patterns of clinker calcined at various temperatures.

The main component of bauxite raw material is diaspore. With the increase in calcination temperature, the diaspore gradually transforms into Al_2_O_3_ crystals. In addition, the raw ore also contains minerals such as kaolinite and chlorite. Figure 4 shows the XRD patterns of the clinker calcined at various temperatures.

Comparing Figure 1 and Figure 4 reveals that the diffraction peak of bauxite diaspore has high intensity after calcination at 500 °C, and the peak shape is sharp. This indicates that the diaspore crystal is intact, and the grain size is relatively large without mineral phase transformation [19]. After calcination at 600 °C, the diffraction intensity suddenly decreases sharply but does not completely disappear, indicating that the diaspore in the bauxite has begun excessive decomposition at 600 °C. After calcination at 700 °C, the diffraction peak of the diaspore completely disappears. This means that it has been completely decomposed and transformed into a corundum phase. The corundum phase in the bauxite exhibits a diffraction peak after calcination at 600 °C, and the diffraction peak intensity increases to a certain extent after calcination at 700 °C. The reason is that kaolinite is dehydrated and transformed into metakaolinite. The corundum diffraction peak intensity does not change significantly with the increase in calcination temperature.

### 2.3. Contrastive Analysis of X-ray Fluorescence Spectra

After bauxite was calcined at various temperatures for 10 min, the chemical composition changed. XRF tests were performed on the clinker calcined at various temperatures to investigate the influence of changes in chemical composition on color changes. Table 2 shows the XRF test results of the bauxite clinker calcined at various temperatures.

As shown in Table 2, after calcination, the Al_2_O_3_ content increases from 60.39% to 68.53% with increasing temperature, indicating a clear upward trend. The content of SiO_2_ increases from 20.36% to 22.83% with the increase in temperature. TiO_2_ exhibits the same descending trend, while K_2_O, Fe_2_O_3_, and MgO remain almost unchanged. It can be seen that the XRF results are consistent with the XRD results.

### 2.4. Contrastive Analysis by Scanning Electron Microscopy

**Figure 5:**
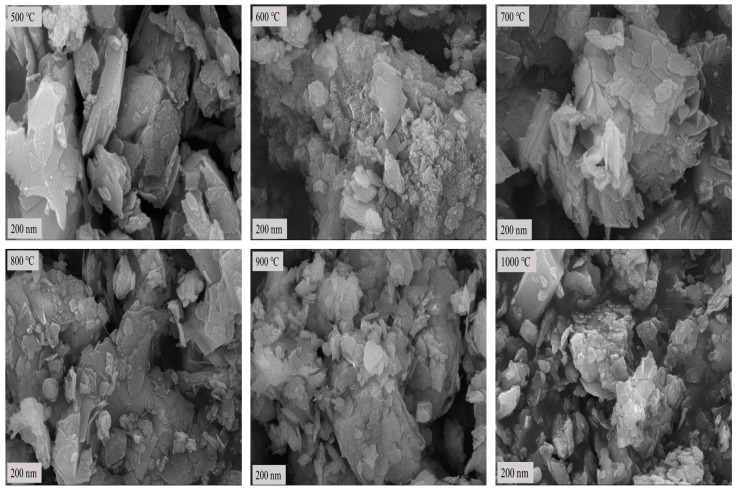
SEM topography of bauxite after calcination at various temperatures.

After calcination at low temperature, SEM was used to observe the bauxite microtopography after calcination at 500 °C, 600 °C, 700 °C, 800 °C, 900 °C, and 1000 °C for 10 min for comparative analysis. Diaspore, the main component of the raw ore, gradually converts to corundum after low-temperature calcination. Therefore, the bauxite morphology changes significantly before and after low-temperature calcination. Figure 5 shows the SEM topography of bauxite after calcination at various temperatures.

Figure 2 and Figure 5 show that the bauxite structure begins to change after calcination at various temperatures for 10 min. At the calcination temperature of 500 °C, the layered structures begin to merge. The reason is that the diaspore loses its structural water, and its crystalline form changes. When the calcination temperature rises to 600 °C, the layered structure of the calcined clinker begins to fade, gradually transforming from the layered structure to granular or columnar corundum crystals. As the calcination temperature rises further, the layered structure of the calcined clinker strengthens binding, which continues to fade. In parallel, the columnar or granular structure and crystallinity continue to increase. This is consistent with the morphological characteristics of corundum crystals.

The clinker calcined at 1000 °C no longer has a clear layered structure, but the granular structure is well developed. This indicates that the product crystallinity reaches its maximum at this point with the densest particle inside [20]. In conclusion, the results of the SEM analysis are consistent with the results of the XRD analysis.

### 2.5. Contrastive Analysis of Fourier Infrared Spectroscopy

**Figure 6:**
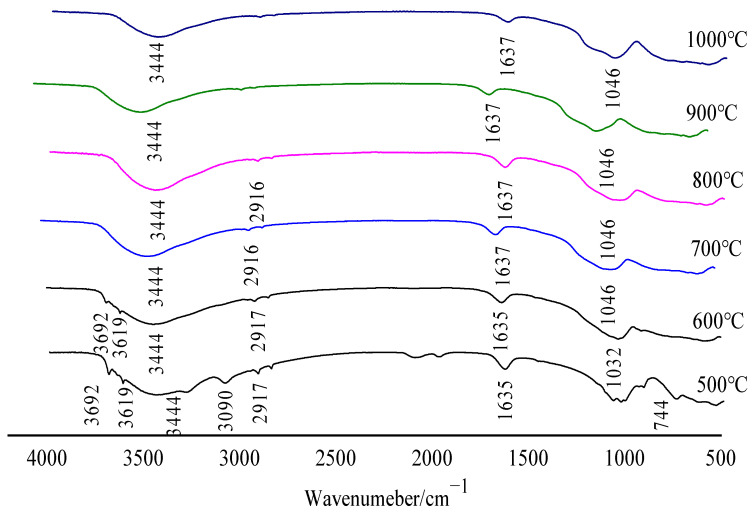
Infrared spectra under different calcination temperatures.

The VERTEX 70 Fourier Infrared Spectrometer from BRUKER (Rheinstetten, Germany) was used to analyze the relationship between the temperature and the gas release products of the sample before and after calcination. As shown in Figure 6, the infrared spectrum of gas release products during oxidative clinker roasting when bauxite was calcined at various temperatures includes three groups: O–H, C–O, and Si–O–T (T = Al, Fe, or Si).

It can be seen from Figure 6 that the absorption spectra of gas products released by bauxite samples during oxidative roasting mainly include O–H, S–O, and C–O groups; according to the mineral composition analysis results of bauxite, the gas products corresponding to the three groups are determined as H_2_O(g), SO_2_, and CO_2_, respectively. Among them, H_2_O(g) is derived from a small amount of free water absorbed by the sample and the dehydroxylation of diaspore, kaolinite, and chlorite. SO_2_ is produced by SO_3_ at high temperature. CO_2_ is produced by the decomposition of CaCO_3_ or the burning of small amounts of organic carbon in the feedstock.

Broadband O–H stretching and O–H bending are observed in the range of 3090–3444 cm^−1^. Fluctuations at 1046 and 3444 cm^−1^ also exist in the infrared spectra after calcination, indicating the presence of water molecules, which are related to the dehydration of bauxite. Combined with TG–DSC analysis, the first wave is caused by the removal of free water in bauxite, and the second wave is caused by the dehydrogenation of diaspore and a small amount of kaolinite and chlorite. The absorption zone in the 2849–2916 cm^−1^ region is associated with C–O bond stretching, which presents the fluctuations caused by the high-temperature decomposition of calcium carbonate in bauxite or the combustion of small amounts of organic carbon. The strongest bands in the 1032–1637 cm^−1^ region correspond to the symmetric stretching vibrations of Si–O–T (T = Al, Fe, or Si). The fluctuation at 744 cm^−1^ corresponds to the S–O bond and the fluctuation is mainly caused by the thermal decomposition of SO_3_.

### 2.6. Comparative Analysis of Organic Carbon Content

**Figure 7:**
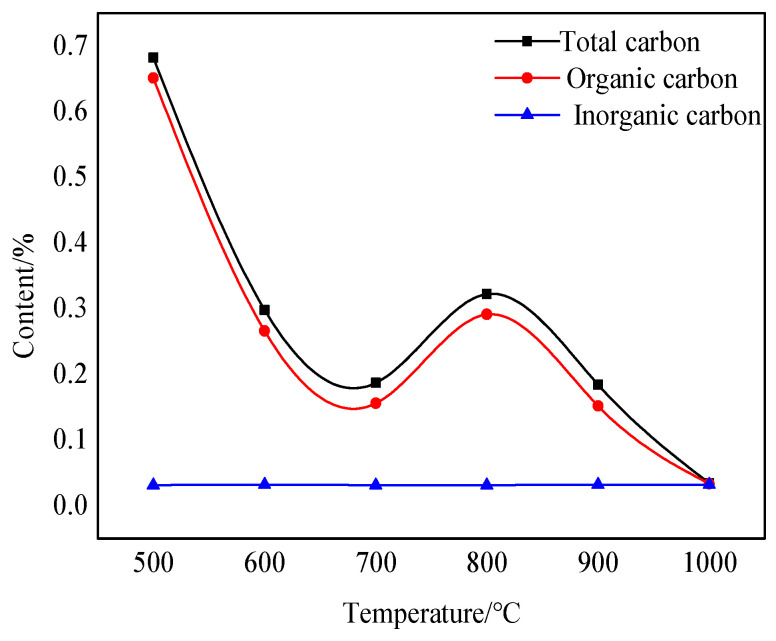
Diagram showing fluctuations in TOC content at various calcination temperatures.

Carbon in nature mainly exists in two forms: organic and inorganic. Although bauxite has a relatively low carbon content, changes in its content also affect the color change after calcination at various temperatures. Figure 7 shows changes in the total carbon and organic carbon content after calcination at various temperatures.

The changing trend in the organic carbon content of the sample is the same as that of the total carbon content after bauxite calcination at various temperatures for 10 min. A noticeable decreasing trend can be seen between 500 °C and 700 °C. The reason is that the organic impurities in bauxite oxidize and burn in this temperature range, turning mainly into carbon-containing gas and volatilizing in the air. There is a period of upward fluctuation between 700 °C and 800 °C. This is caused by the carbonization of only some organic impurities at high temperatures. Throughout the calcination process, temperature changes have little effect on the inorganic carbon content.

### 2.7. Image Color Feature Extraction

**Figure 8:**
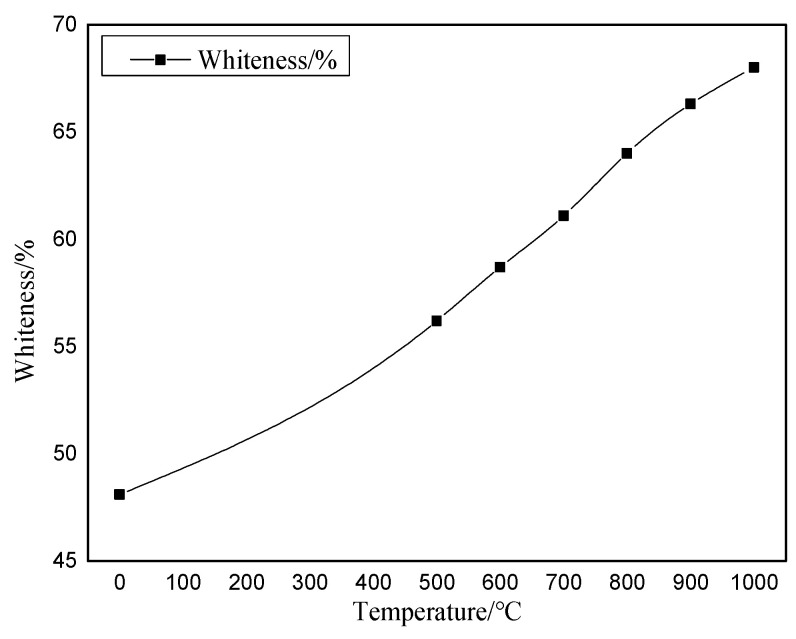
Diagram of whiteness change at various calcination temperatures.

**Figure 9:**
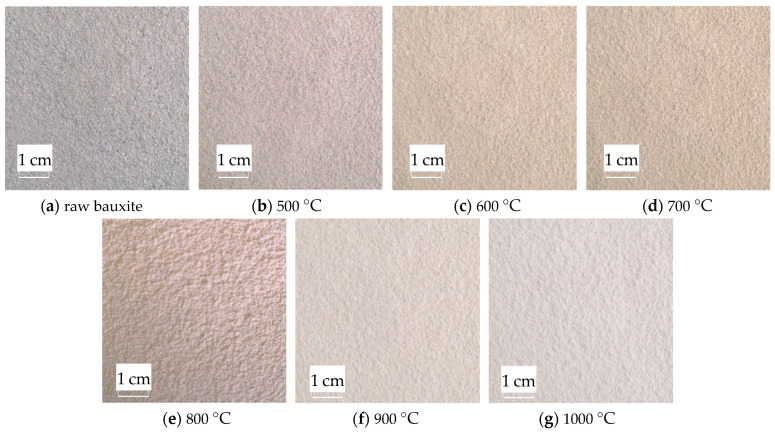
Images after calcination at various temperatures.

After the bauxite is calcined at various temperatures for 10 min, the macroscopic appearance of the color change is the change in whiteness. Using the WSB-X digital whiteness meter from Sichuan Sichuang Beike Technology Co., Ltd. (Suining, China), the sample whiteness is observed after calcination at various temperatures. The whiteness variation range is shown in Figure 8. The original color image after calcination at various temperatures is shown in Figure 9.

As shown in Figure 8 and Figure 9, bauxite exhibits noticeable changes in color characteristics after calcination at various temperatures for 10 min. As the calcination temperature rises from 500 °C to 1000 °C, the whiteness of the clinker rises from 56.2% to 68%. This indicates that the bauxite color change after calcination is a gradual whitening process.

**Figure 10:**
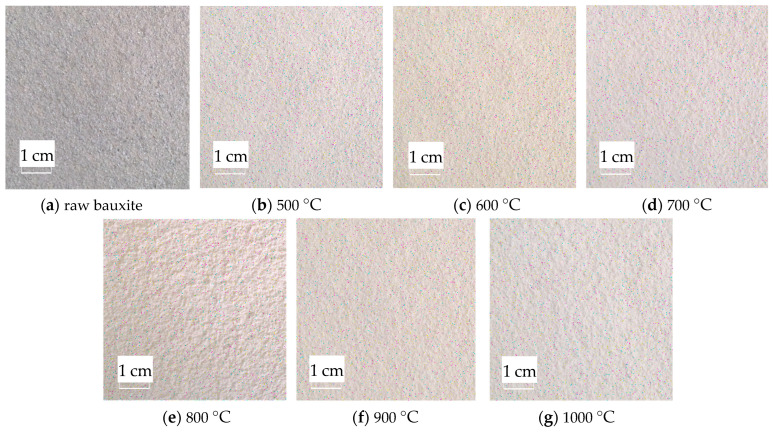
Median filter diagram of the original image.

After calcination, colored bauxite images are obtained and preprocessed using a median filter. The median filter effect diagram is shown in Figure 10.

Figure 10 shows that after adding salt and pepper noise with a mean value of 0 and a variance of 0.3 to the images calcined at various temperatures, the median filter is used to denoise with a satisfactory denoising effect. After filtering, the image blurring is reduced, the noise is reduced, and the image integrity is well preserved. This provides an excellent basis for rapidly extracting subsequent color features.

**Figure 11:**
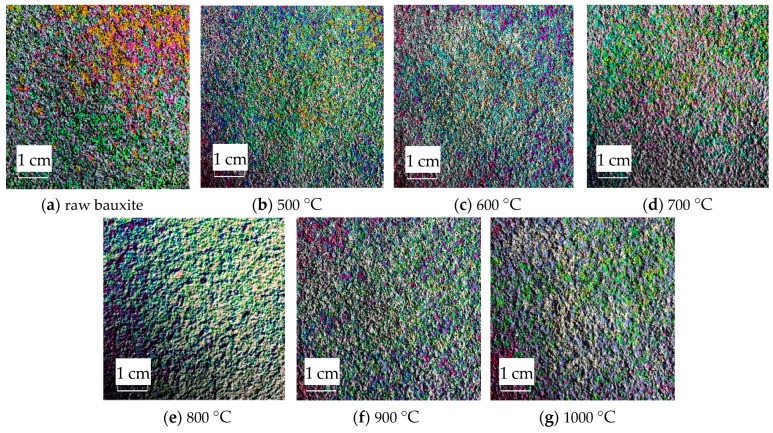
Equalized images of the HSV histogram of original images.

**Figure 12:**
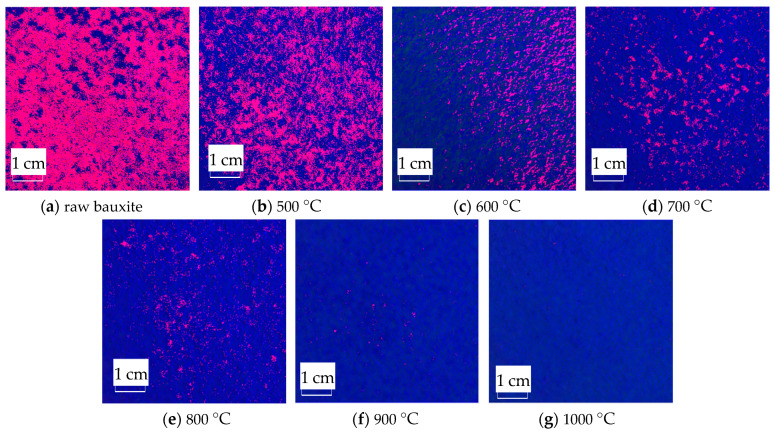
HSV conversion diagram of images after calcination at various temperatures.

**Figure 13:**
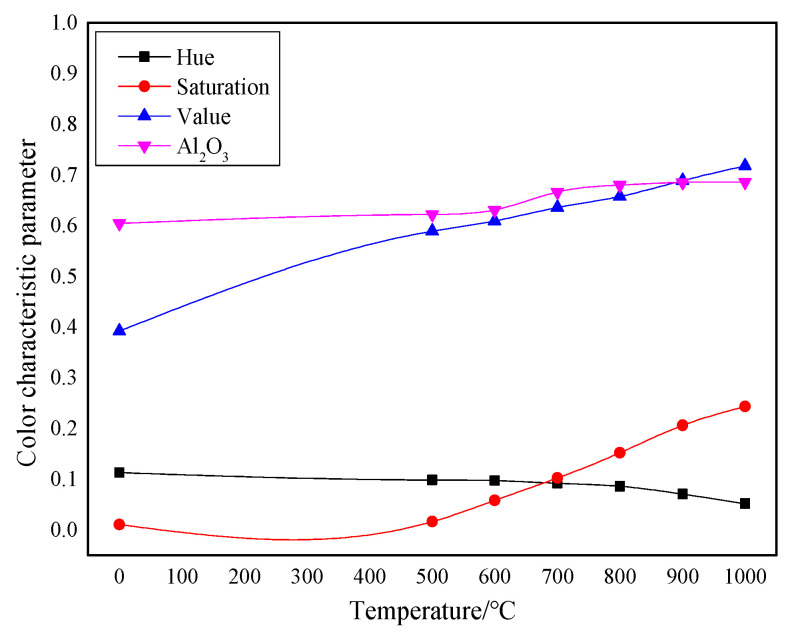
Comparison of color characteristics and Al_2_O_3_ content after calcination at various temperatures.

The RGB image after HSV histogram equalization is shown in Figure 11, and the HSV conversion diagram obtained is shown in Figure 12. After processing by MATLAB 2019a, the specific parameters of the three components H, S, and V are obtained. Figure 13 shows the changes in parameters H, S, and V and the changes in the Al_2_O_3_ content of bauxite during the calcination process.

As shown in Figure 11, after the original RGB image is histogram-equalized in the H, S, and V color spaces, all pixels of the original image are mapped to the equalized image. Compared to RGB color space images, equalized images can enhance the overall image contrast effect without changing the image information structure.

The HSV conversion diagram reflects the intuitiveness of colors [21], which coincides with the human visual perceived color. Figure 12 shows the HSV conversion diagram. The observation indicates that the bauxite raw material image is basically red [22]. Increasing the calcination temperature will gradually reduce the red areas in the image. This trend is consistent with the whiteness variation. As shown in Figure 13, the color characteristic value parameters of bauxite show a relatively obvious variation under various calcination temperatures. Under a calcination time of 10 min, when the temperature rises from 500 °C to 1000 °C, the brightness characteristic value that the human eye recognizes as the brightness of a light source or an object and the saturation characteristic value that indicates color purity tend to increase noticeably. The chromaticity characteristic value that reflects color characteristics is on a downward trend. The trend of the change in Al_2_O_3_ content after calcination at various temperatures is consistent with the changing trend in the brightness and saturation.

## 3. Sampling and Methods

### 3.1. Sample Preparation

The bauxite used in the experiments was taken from a bauxite mine in Xiuwen District, Guizhou Province, and a total of 50 kg of ore samples were taken using the random sampling method. The samples were blue-gray in appearance, lumpy in shape, and belonged to the monohydrate type (D-K type). Considering the large volume of massive ore and a certain amount of impurities attached to the surface, the ore was first screened and washed to ensure test accuracy. It was then dried in a 101-AS electrothermal blowing dry box and finally ground into a powdered bauxite sample (through a 200-mesh sieve) of the particle size required for the test.

### 3.2. Test Methods

(1) Low-temperature calcination of raw bauxite

According to the preliminary exploration test, the test conditions were determined: raw bauxite was crushed to 200 mesh. To ensure uniform sample heating, 10 g of sample was taken each time and placed into a ceramic crucible. The ceramic fiber furnace model TC-2.5-12 was heated at a step of 10 °C/min at room temperature. The ceramic crucible containing the sample was calcined for 10 min when the temperature reached 500 °C, 600 °C, 700 °C, 800 °C, 900 °C, and 1000 °C. After the calcination ended, the sample was taken out of the ceramic fiber furnace and placed in a drying dish to cool until room temperature.

(2) Microscopic compositional analysis

The XRD POWDER PRO X-ray diffractometer produced by PANalytical B.V (Almelo, The Netherlands). was used to analyze the crystal phases of bauxite before and after the calcination. The CuKα ray source (λ = 0.154 nm) was used. The tube voltage was 40 kV and the tube current was 40 mA. The process adopted a step scan with a step length of 0.05 and a scan speed of 0.5 o/s.

The structural water and bound water of bauxite were analyzed with the STA2500 Regulus Synchronous Thermal Analyzer (TG–DSC) from NETZSCH (Selb, Germany). The heating range was 1000 °C and the heating rate was 10 °C/min under an air atmosphere.

The ARL Peform’X wavelength-dispersive X-ray fluorescence spectrometer produced by Thermo Fisher Scientific (Waltham, MA, USA) was used to analyze the chemical composition of bauxite before and after calcination. During the test, 4.0 g of samples was accurately weighed (dried and sieved through a 200 mesh) and placed into a mold. The bottom was filled with boric acid powder, pressurized at 40 T, and held for 30 s to create a disk with a sample diameter of 29 mm. It was then placed in the corresponding sample cup of the instrument, and UniQuant semi-quantitative software (https://assets.thermofisher.cn/TFS-Assets%2FMSD%2FSpecification-Sheets%2FXR-PS41207-UniQuant.pdf) was used for analysis in a vacuum environment. The gas used by the detector was P10 gas (10% methane and 90% argon), and the analysis was run under the following conditions: power ≥ 4.2 kW, voltage ≥ 60 kV, current ≥ 120 mA, and Be window thickness of 75 m. The target material used for the test was a rhodium target, and the crystals used for the analysis were LiF200 (60 kV, 40 mA), LiF220 (60 kV, 40 mA), Ge111 (40 kV, 60 mA), and AX03 (30 kV, 80 mA).

The VERTEX 70 Fourier Infrared Spectrometer from BRUKER (Karlsruhe, Germany) was used to analyze the relationship between the temperature and the gas release products of the sample before and after calcination. A small amount of sample (about 0.6 mg) and a certain amount of potassium bromide (about 200 mg) were placed in an agate mortar for uniform grinding and tableting. A wavenumber range of 4000 to 400 cm^−1^ was selected to perform the FT-IR test on the sample.

The S-3400 scanning electron microscope produced by Tianli (Tōkyō, Japan) with a working voltage of 20 kV was used to detect changes in the surface structure and crystalline state of the sample before and after calcination at various temperatures.

The WSB-X digital whiteness meter from Sichuan Sichuang Beike Technology Co., Ltd. was used to detect the whiteness of samples calcined at various temperatures under natural indoor conditions.

The change in organic carbon content before and after calcination was determined using an organic element analyzer (FLASH 2000 CHNS/O, Semofei Company, Waltham, MA, USA). During the test, ~1 g of the sample was taken and dried at 50 °C for 24 h. After drying, the sample was taken out and weighed in a dryer. The sample was ground to 200 mesh, and 1 mol/L of hydrochloric acid was added according to the content of calcium carbonate. After stirring with a magnetic agitator until the carbonate was completely reacted, it stood for 24 h. Pure water was added repeatedly for cleaning and centrifugation, and the supernatant was removed and detected with pH test paper; this was repeated until a neutral pH was obtained. After drying for 24 h, the sample was put on the machine for testing.

(3) Image color feature extraction

**Figure 14:**
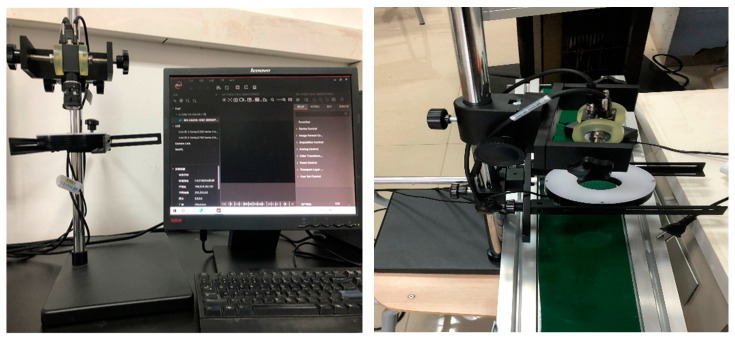
Image acquisition system.

The image acquisition system used in this paper is composed of an MV-CA050-10GC industrial camera (5 MP, COMS sensor) produced by Hikvision (Hangzhou, China), a Lenovo Y7000p computer, and an auxiliary light source. In order to reduce the error caused by environmental factors, the machine vision system was set up with an independent ring light source system and configured with a digital light control system and a light shield to isolate the test system from external light sources. At the same time, a filter lens was set on the lens, and dust cleaning was carried out with special tools after each test. After collecting bauxite images calcined at different temperatures, the images were first grayed out and median-filtered to denoise. The image RGB color space was converted to HSV space and finally processed by MATLAB 2019, a software package used to extract image color feature parameters (chromaticity, brightness, saturation) [23]. The image acquisition system is shown in Figure 14.

During image processing, the process of reducing noise in digital images is called image denoising. This paper used median filtering after adding salt and pepper noise for image denoising. Its basic principle is to replace the pixel value of a certain point in a digital sequence or image with the median value of each point in the neighborhood. Its definition is shown in Equation (4):

(4) $$y=med(x_{1},x_{2},\ldots x_{n})=\left\{\begin{array}{c} x_{i\frac{n+1}{2}}\\ \frac{1}{2}\left[x_{i\frac{n+1}{2}}+x_{i(\left(\frac{n}{2}+1)\right)}\right] \end{array}\right.$$

The histogram equalization of the image is also known as histogram flattening. There is a visual effect of rough classification under a small output data segment value of the equalized histogram. Equalization of the image histogram facilitates digital image processing, and its function expression is shown in Equation (5):

(5) $$S_{i}=T(r_{i})=\sum_{i-0}^{k-1}\frac{n_{i}}{n}$$

where *k* is the number of gray levels.

Currently, the most commonly used color space in the color feature extraction process is the HSV space. The distance between two color points in the HSV space represents the distance between the two colors. This distance can adequately define the distance between the two colors recognized by the human eye [24], which is in line with human perception psychology. By quantifying the space, the extracted color features match the visual characteristics, and the similarity (color distance) matches the visual similarity. Images collected by industrial cameras are RGB images. Therefore, in order to obtain the corresponding three components of H, S, and V, it is necessary to move the RGB images into the HSV color space before performing subsequent image processing. Equations (6)–(9) show the conversion process:

(6) $$V=\frac{1}{\sqrt{3}}(R+G+B)$$

(7) $$S=1-\frac{\sqrt{3}}{V}min(R,G,B)$$

(8) $$H=\left\{\begin{array}{c} \theta \;G\geq B\\ 2\pi -\theta \;G<B \end{array}\right.$$

(9) $$\theta =\cos^{-1}\left[\frac{\frac{1}{2}\left[\left(R-G)+(R-B\right)\right]}{\sqrt{{\left(R-B)(G-B)+(R-G\right)}^{2}}}\right]$$

## 4. Conclusions

To improve the detection rate of calcination effects during the low-temperature calcination of DK bauxite from Guizhou, large equipment such as XRD was used to analyze the micro-components of bauxite after calcination at various temperatures. Next, a machine-based visual image feature extraction technique was used to extract the image color features after calcination at various temperatures to detect the bauxite calcination effect quickly. In this research, the main conclusions are drawn as follows:

(1) Tested by instrumental analysis suggest the following microscopic evidence for the bauxite color change from Guizhou after calcination. As the calcination temperature rises from 500 °C to 1000 °C, diaspore (the main bauxite component) is gradually transformed into Al_2_O_3_ at high temperatures. Free water, weakly bound water on the surface, and chemically adsorbed bound water are sequentially dehydrated. Finally, trace organic carbon is gradually removed by oxidation and combustion.

(2) Using the feature extraction method based on the HSV color space, image color feature parameters were derived after bauxite calcination at various temperatures. When calcining from 500 °C to 1000 °C, the chromaticity value (H) of the image dropped from 0.0980 to 0.0515, the saturation value (S) rose from 0.0161 to 0.2433, and the brightness value (V) rose from 0.5890 to 0.7177.

(3) The color characteristics of the image of this bauxite after low-temperature calcination had more obvious changes after calcination at 500–1000 °C: the chromaticity value (H) of the image decreased from 0.1130 in the original ore to 0.0515 after calcination at 1000 °C; the saturation (S) increased from 0.0104 in the original ore to 0.2433 after calcination at 1000 °C; and the brightness (V) increased from 0.3920 in the original ore to 0.7177 after calcination at 1000 °C. The corresponding Al_2_O_3_ content increased from 60.39% in the original ore to 68.53% after calcination at 1000 °C. The correlation between color characteristics and Al_2_O_3_ content provides a new method for determining the calcination effect of bauxite in Guizhou.

## Figures and Tables

**Table 1:** Main chemical constituents of bauxite.

Component	Al_2_O_3_	SiO_2_	TiO_2_	K_2_O	Fe_2_O_3_	MgO	SO_3_	CaO	P_2_O_5_	ZrO_2_	Else	LOI
Content (%)	60.39	19.17	2.84	1.03	1.08	0.77	0.14	0.16	0.10	0.05	0.51	13.76

**Table 2:** XRF test results of bauxite clinker calcined at various temperatures.

	Content/%	Al_2_O_3_	SiO_2_	TiO_2_	K_2_O	Fe_2_O_3_	MgO	CaO	P_2_O_5_	ZrO_2_	Else
Component											
500 °C		62.18	20.36	3.16	1.41	1.18	0.87	0.19	0.11	0.07	6.47
600 °C		63.08	20.67	3.25	1.43	1.28	0.88	0.20	0.12	0.08	5.01
700 °C		66.59	21.04	3.47	1.46	1.29	0.92	0.22	0.13	0.09	2.79
800 °C		67.99	22.62	3.55	1.49	1.31	0.93	0.23	0.15	0.09	1.64
900 °C		68.51	22.75	3.58	1.51	1.34	0.94	0.24	0.17	0.09	0.87
1000 °C		68.53	22.83	3.68	1.52	1.35	0.95	0.25	0.18	0.1	0.61

## Data Availability

Data are contained within the article.
